# Early antibiotic review and AWaRe prescribing in respiratory infections before vs during COVID-19: a two-hospital retrospective cohort study

**DOI:** 10.1080/20523211.2026.2719528

**Published:** 2026-09-14

**Authors:** Rasha Abdelsalam Elshenawy, Nkiruka Umaru, Zoe Aslanpour

**Affiliations:** Department of Medicine, School of Health, Medicine, and Life Sciences, University of Hertfordshire, Hatfield, UK

**Keywords:** Antimicrobial stewardship, antibiotic review, COVID-19, AWaRe classification, start smart-then focus, CARES framework, secondary care, respiratory tract infections

## Abstract

**Background:**

Antimicrobial stewardship (AMS) is essential for optimising antibiotic use and limiting antimicrobial resistance. The COVID-19 pandemic disrupted healthcare delivery, but its effect on early antibiotic review remains unclear. This study compared antibiotic review practices and prescribing patterns among hospitalised patients with respiratory tract infections before and during the pandemic.

**Methods:**

A retrospective cohort study was conducted across two acute hospitals within an NHS Foundation Trust in England. Adults aged 25 years or older admitted with respiratory tract infections and prescribed systemic antibiotics were included from 2019 (pre-pandemic) and 2020 (pandemic). Data covered review timing, stewardship interventions based on the CARES framework, and prescribing patterns according to the WHO AWaRe classification. Stratified systematic random sampling identified 640 patients: 320 per year and 160 per hospital annually.

**Results:**

Median age was 78 years in 2019 and 79 years in 2020. Mortality was comparable (15.0% versus 15.6%; *p* = 0.83). Early antibiotic review at 48–72 hours was maintained across both periods (51.6% versus 50.9%; *p* = 0.87). However, Watch antibiotic prescribing increased significantly from 52% to 60% (*p* < 0.001), accompanied by reduced Access antibiotic use, while Reserve use remained restricted (3–5%). Among guideline-concordant prescriptions, de-escalation was the most frequent intervention (19–38%). Continuation without documented change increased from 7% and 9% in 2019 to 21% and 16% in 2020 at Hospitals A and B, respectively.

**Conclusions:**

Early antibiotic review remained resilient during COVID-19 despite operational pressures. Nevertheless, increased Watch prescribing highlights the need for enhanced diagnostic stewardship and pandemic-resilient AMS infrastructure. Embedded review processes, multidisciplinary collaboration, workforce development, and digital stewardship tools may strengthen antibiotic optimisation during future healthcare disruptions. The contribution of pharmacists requires further investigation. These findings demonstrate that maintaining review frequency alone may not prevent broader-spectrum prescribing and support targeted strategies to improve documentation, promote Access antibiotics, and preserve stewardship quality during periods of pressure.

## Introduction

Antimicrobial resistance (AMR) represents one of the most pressing global health threats, with antibiotic-resistant infections directly causing 1.27 million deaths in 2019 and contributing to 4.95 million deaths worldwide (Antimicrobial Resistance Collaborators, 2022; Naghavi et al., 2024; WHO, 2023). Projections suggest AMR could cause up–10 million annual deaths by 2050 if current trends continue (De Kraker et al., 2016; Price, 2016). Antimicrobial stewardship (AMS), a systematic approach to optimising antibiotic prescribing, is central to mitigating this threat by improving patient outcomes, reducing adverse events, and preserving antibiotic efficacy (Abdelsalam Elshenawy, Umaru, and Aslanpour, 2023; Khadse et al., 2023; NICE, 2015).

A cornerstone of effective AMS is the timely review of antibiotic therapy following initiation. International guidelines recommend that antibiotics be reviewed within 48–72 h of commencement, when diagnostic results are typically available, and the clinical response can be assessed (Chung et al., 2013; UK Health Security Agency, 2024). This review enables key stewardship interventions, including de-escalation to narrower-spectrum agents, switching from intravenous to oral therapy, and discontinuation of unnecessary therapy (BSAC, 2018; Department of Health and Social Care, 2024). In the United Kingdom, the UK Health Security Agency's ‘Start Smart – Then Focus’ (SSTF) framework operationalises this approach through structured review outcomes (UKHSA, 2024). At the same time, the World Health Organisation's AWaRe classification system provides a tool for monitoring antibiotic consumption patterns based on resistance risk (Abdelsalam Elshenawy, Umaru, Alharbi, et al., 2023; WHO, 2023). Despite these established frameworks, evidence on optimal review timing and its relationship with stewardship outcomes in real-world hospital settings remains limited (Giamarellou et al., 2023).

Pharmacists are increasingly recognised as central to delivering effective antimicrobial stewardship. A cross-sectional survey conducted at the same NHS Foundation Trust during this study period found that 85.2% of pharmacists recognised AMR as a public health concern, 91.2% believed actions against AMR would benefit society, and 85.6% supported AMS for prudent antibiotic use, demonstrating strong workforce capacity for stewardship delivery (Abdelsalam et al., 2025).

During 2020, the COVID-19 pandemic significantly disrupted AMS implementation globally (Abdelsalam Elshenawy et al., 2025; Li et al., 2024). Despite guidance discouraging antibiotic use without confirmed bacterial co-infection, antimicrobials were administered to approximately 70% of hospitalised COVID-19 patients, with marked increases in broad-spectrum (Watch category) antibiotic prescribing (Abdelsalam Elshenawy, Umaru, Alharbi, et al., 2023). Operational pressures, including delayed microbiological testing, reduced multidisciplinary team engagement, and competing clinical priorities, further challenged the maintenance of timely antibiotic review practices (Wang et al., 2025; WHO, 2024). However, whether early review practices and stewardship interventions were sustained during this period of unprecedented healthcare disruption remains unclear (Pierce & Stevens, 2021).

This retrospective cohort study aimed to evaluate antibiotic review practices in adult patients with respiratory tract infections at two UK secondary care hospitals in 2019 and 2020. Specifically, we sought to: (1) characterise the timing of antibiotic reviews and associated stewardship interventions; (2) describe antibiotic prescribing patterns using the WHO AWaRe classification; and (3) identify opportunities for strengthening AMS resilience during public health emergencies.

## Methods

### Study design and setting

This retrospective cohort study was conducted at a single NHS Foundation Trust in England, comprising two acute care hospitals (Hospital A and Hospital B) in the East of England. The Trust serves a population of approximately 400,000 people and has around 742 inpatient beds. The study evaluated antibiotic review practices, prescribing patterns, and AMS interventions in adult patients admitted with respiratory tract infections (RTIs) during two time periods: 2019 and 2020. Both hospitals operated under the same Trust-wide antimicrobial guidelines, formulary, and electronic prescribing system throughout the study period.

### Study population and eligibility criteria

The study included adults aged 25 years and older who were admitted to general medical, respiratory, or acute medical wards and prescribed systemic antibiotics for RTIs. Patients were identified using International Classification of Diseases, 10th Revision (ICD-10) codes for respiratory infections (Supplemental Table S1). Eligible diagnoses included community-acquired pneumonia (CAP), hospital-acquired pneumonia (HAP), acute exacerbation of chronic obstructive pulmonary disease (COPD), lower respiratory tract infection (LRTI), upper respiratory tract infection (URTI), and COVID-19 pneumonia (2020 only).

Patients were excluded if they: (1) were aged under 25 years; (2) were not prescribed antibiotics during admission; and (3) had a hospital stay of less than 48 h (insufficient time for antibiotic review). The age threshold of 25 years was selected to reflect the adult general medical population admitted with respiratory infections at this Trust, excluding younger adults whose presentations and empirical treatment pathways differ. It is acknowledged that excluding patients with stays <48 h may introduce selection bias towards more complex or severe admissions, which could influence prescribing patterns; this is noted as a study limitation. Pregnant and immunocompromised patients were included to reflect real-world prescribing practice.

### Sample size calculation

Sample size was calculated using the single-proportion formula [n = z²P(1−P)/d²], implemented in Minitab, where z = 1.96 (95% confidence level), *P* = 0.20 (estimated inappropriate prescribing rate, based on national surveillance data from Public Health England, 2018), and d = 0.10 (margin of error), yielding a required sample of 640 patients (Minitab, 2021). Equal allocation across strata (320 per year; 160 per hospital per year) was selected to ensure balanced seasonal and hospital representation and to facilitate direct pre-pandemic versus pandemic comparisons.

### Sampling strategy

A stratified systematic random sampling approach was employed. Eight time points were defined: March, June, September, and December of 2019 and 2020, with 80 patients sampled per time point (40 per hospital). These periods were selected to capture seasonal variation and key pandemic phases, including the first wave (March–June 2020), the national lockdown, the second wave (September–December 2020), and the early vaccination rollout (noting that this phase was applicable only to the December 2020 stratum, when the NHS vaccination programme had recently commenced, and its direct influence on prescribing was not assessed in this study).

**Figure 1.**
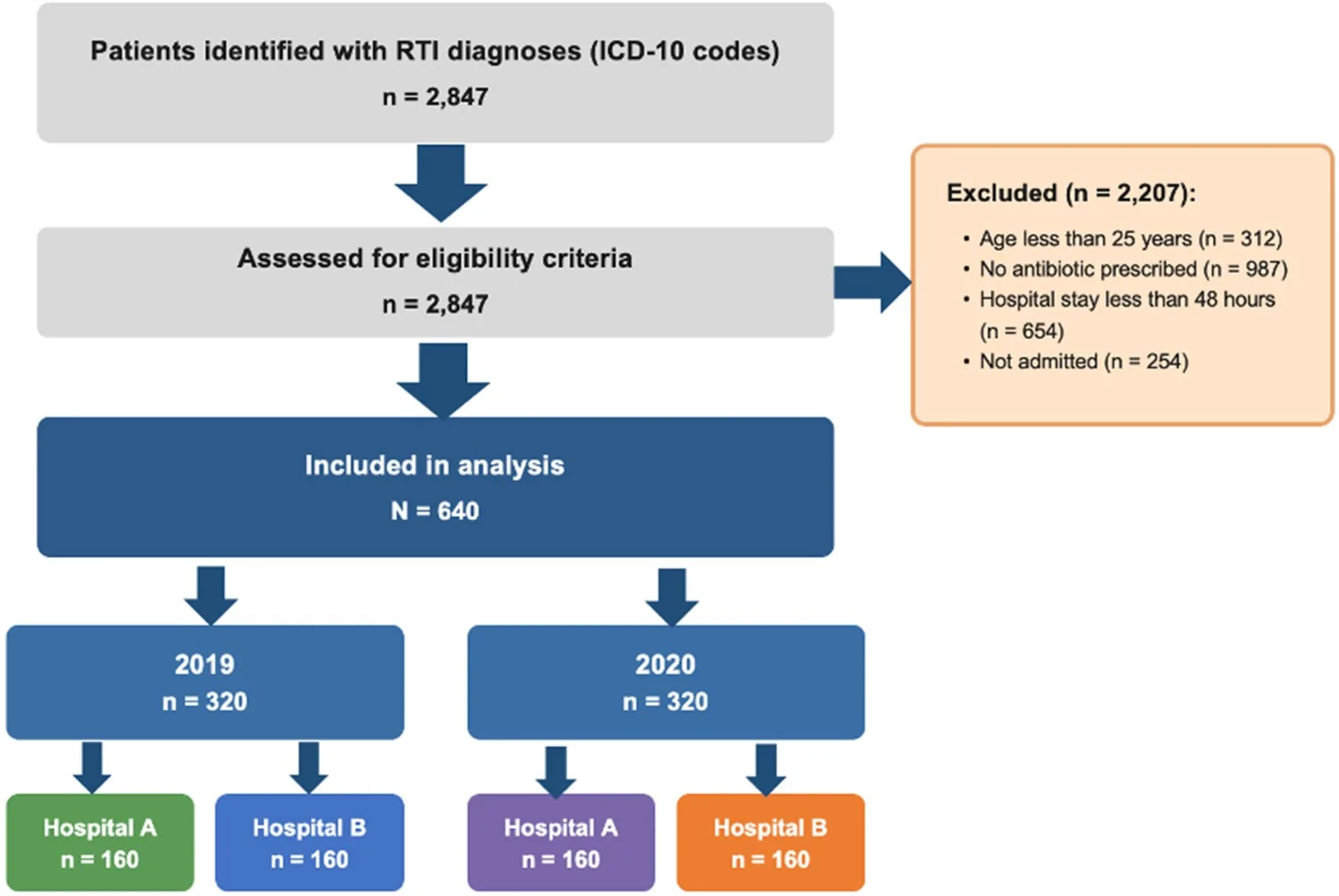
Patient selection flow diagram (STROBE). Note: Patient selection flow diagram prepared in accordance with the STROBE statement. Of 2,847 patients identified with respiratory tract infection (RTI) diagnoses by ICD-10 codes, 2,207 were excluded (age <25 years, *n* = 312; no antibiotic prescribed, *n* = 987; hospital stay <48 h, *n* = 654; not admitted, *n* = 254), yielding 640 patients included in the analysis (320 per year; 160 per hospital per year). Abbreviations: RTI, respiratory tract infection; ICD-10, International Classification of Diseases, 10th Revision.

Within each stratum, all patients meeting ICD-10 inclusion criteria were identified from electronic health records and assigned sequential identification numbers (World Health Organisation, 2019). A computer-generated random number sequence was used to select patients until the target of 40 per hospital per time point was achieved (Haahr, 2019). This systematic random approach ensured representativeness while minimising selection bias. A patient flow diagram illustrating the selection process is provided in Figure 1.

### Data sources and extraction

Data were extracted from the Trust's electronic health records by the primary author, using a standardised data extraction tool developed specifically for this study. Each patient record required approximately 45 min to review. Extracted variables included demographic data, clinical characteristics, prescribing data, and outcomes (Supplemental Table S2), as outlined below:

- **Demographics:** Age, sex.
- **Clinical characteristics:** Primary diagnosis, comorbidities, and documented drug allergies.
- **Prescribing data:** Initial empirical antibiotic, antibiotic class (WHO AWaRe classification: Access, Watch, Reserve), and route of administration (WHO, 2023).
- **Diagnostic findings:** Chest radiograph results, categorised as pneumonia confirmed, no pneumonia, or not performed.
- **Comorbidities:** Defined as follows – hypertension (documented diagnosis or antihypertensive therapy); diabetes mellitus (type 1 or type 2); chronic kidney disease (stages 3–5) or acute kidney injury; chronic liver disease (including cirrhosis, hepatitis, or fatty liver disease); heart failure (documented diagnosis with or without reduced ejection fraction); malignancy (active cancer); and chronic obstructive pulmonary disease.
- **Antibiotic review timing:** Categorised as Day 2–3, Day 4, or Day 7 post-initiation. Review timing was defined as the interval between antibiotic initiation and a documented clinical review resulting in a prescribing decision. Reviews were classified as early (Day 2–3), intermediate (Day 4), or late (Day 7), in alignment with the UK Health Security Agency (UKHSA) Start Smart – Then Focus framework.
- **Antimicrobial stewardship interventions:** Classified according to the UKHSA Antimicrobial Stewardship Toolkit CARES outcomes (Cease, Amend, Review, Escalate, Switch) as: cessation (antibiotic stopped), de-escalation (switch to a narrower-spectrum agent), escalation (switch to a broader-spectrum agent), intravenous-to-oral switch, continuation (no change with documented review), or other change (UKHSA, 2024).
- **Appropriate prescribing:** Defined as concordance with the Trust's local antimicrobial guidelines for empirical therapy, based on clinical indication, infection severity, patient-specific factors (including allergies and renal function), and documented clinical rationale. Prescriptions were classified as appropriate or inappropriate by comparison with guideline recommendations current at the time of prescribing.
- **Outcomes:** Discharge from hospital or in-hospital death.

### Data quality and validation

A pilot study of 80 patient records was conducted to assess feasibility and refine the data extraction tool prior to full data collection. For validation, two authors (RAE and NU) independently extracted data from a randomly selected 5% sample (*n* = 32 records). Inter-rater reliability was assessed by calculating percentage agreement and Cohen's kappa coefficient for key categorical variables. Observed agreement was 92% overall. Cohen's kappa values were: antibiotic classification (κ = 0.89), review timing (κ = 0.91), stewardship intervention type (κ = 0.86), and appropriateness classification (κ = 0.84), indicating substantial to almost perfect agreement. Discrepancies were resolved through discussion and consensus, and the data extraction protocol was clarified accordingly.

### Statistical analysis

Descriptive statistics were used to summarise patient characteristics, prescribing patterns, and stewardship interventions. Categorical variables were presented as frequencies and percentages. Comparisons between hospitals and between study periods were performed using chi-square tests (or Fisher's exact test where expected cell counts were <5) for categorical variables and Mann–Whitney U tests for continuous variables. Statistical significance was set at *p* < 0.05 (two-tailed). All analyses were performed using IBM SPSS Statistics version 22.0 (SPSS, 2020).

### Ethical considerations and reporting

The study was approved by the Health Research Authority (HRA) and the Research Ethics Committee (REC reference: 22/EM/0161). Institutional approval was granted by the University of Hertfordshire Ethics Committee (reference: LMS/PGR/NHS/02975). The study is reported in accordance with the Strengthening the Reporting of Observational Studies in Epidemiology (STROBE) guidelines (STROBE, 2023).

### Patient and public involvement

The study protocol was reviewed by the Citizens Senate, a patient and public involvement organisation with substantial representation of elderly individuals. Feedback informed the study design and interpretation of findings, particularly regarding the relevance of outcomes to patient care.

### Study registration

This study was prospectively registered with the ISRCTN registry (ISRCTN14825813) and Octopus, the global primary research record (ISRCTN, 2019; OCTOPUS, 2020). Registration was undertaken prospectively because this manuscript forms Phase 1 of a two-phase research project. Phase 1 comprises the retrospective medical record review reported here (*n* = 640 patients), and Phase 2 comprises a prospective cross-sectional survey of healthcare professionals (*n* = 240) at the same NHS Foundation Trust. The ISRCTN registration covers the full research project, and the protocol is aligned with the methodology reported across both phases.

## Results

### Study population

A total of 640 patients were included in the study, with 320 patients in each study period (2019 pre-pandemic and 2020 pandemic). Each hospital contributed 160 patients per year. The patient selection process is illustrated in Figure 1.

### Demographic and clinical characteristics

Table 1 presents the demographic characteristics, clinical indications, and comorbidities of the study population. The median age was 78 years (IQR 66–85) in 2019 and 79 years (IQR 67–87) in 2020. The sex distribution was balanced, with 321 males (50.2%) and 319 females (49.8%) across the full cohort. In-hospital mortality was similar between periods: 48 patients (15.0%) in 2019 and 50 patients (15.6%) in 2020 (*p* = 0.83). Documented drug allergies were present in 53 patients (8.3%).

**Table 1.** Demographic characteristics, clinical indications, comorbidities, and outcomes of patients with respiratory tract infections (*N* = 640).

Variable	2019 (*n* = 320)	2020 (*n* = 320)	*p*-value
Age, median (IQR)	78 (66–85)	79 (67–87)	0.24
**Sex**			0.49
Male	156 (48.8)	165 (51.6)	
Female	164 (51.2)	155 (48.4)	
**Patient outcome**			0.83
Discharged	272 (85.0)	270 (84.4)	
Died	48 (15.0)	50 (15.6)	
Documented allergy	26 (8.1)	27 (8.4)	0.89
**Clinical indication**			
CAP	126 (39.4)	136 (42.5)	0.42
HAP	67 (20.9)	52 (16.2)	0.12
COPD exacerbation	30 (9.4)	14 (4.4)	**0**.**01**
LRTI	30 (9.4)	23 (7.2)	0.31
Unspecified pneumonia	56 (17.5)	42 (13.1)	0.12
COVID-19 pneumonia	0 (0.0)	44 (13.8)	**<0**.**001**
URTI	6 (1.9)	8 (2.5)	0.59
VAP	5 (1.6)	1 (0.3)	0.10
**Comorbidities**			
Hypertension	143 (44.7)	148 (46.3)	0.69
Atrial fibrillation	61 (19.1)	64 (20.0)	0.77
Heart failure	32 (10.0)	63 (19.7)	**<0**.**001**
Diabetes mellitus	65 (20.3)	54 (16.9)	0.27
Chronic kidney disease	75 (23.4)	46 (14.4)	**0**.**003**
COPD	42 (13.1)	40 (12.5)	0.82
Malignancy	50 (15.6)	43 (13.4)	0.43
Asthma	35 (10.9)	21 (6.6)	**0**.**05**
Dementia	25 (7.8)	23 (7.2)	0.77
Liver disease	8 (2.5)	19 (5.9)	**0**.**03**
Depression	12 (3.8)	20 (6.3)	0.14

Note: Data presented as n (%) unless otherwise specified. CA*P* = community-acquired pneumonia; HA*P* = hospital-acquired pneumonia; COPD = chronic obstructive pulmonary disease; LRTI = lower respiratory tract infection; URTI = upper respiratory tract infection; VA*P* = ventilator-associated pneumonia; IQR = interquartile range. Chronic kidney disease is defined as eGFR <60 mL/min/1.73m² or renal replacement therapy. Liver disease includes cirrhosis, chronic hepatitis, and non-alcoholic fatty liver disease. Malignancy is defined as active cancer or a diagnosis within the preceding 5 years. *P*-values from chi-square tests (categorical) or Mann-Whitney U test (continuous). Comorbidities do not sum to 100% as patients may have multiple conditions.

Community-acquired pneumonia (CAP) was the most common indication for antibiotic therapy, accounting for 126 cases (39.4%) in 2019 and 136 cases (42.5%) in 2020. Hospital-acquired pneumonia (HAP) was the second most frequent indication (67 cases [20.9%] in 2019; 52 cases [16.2%] in 2020). COVID-19 pneumonia emerged as a new indication in 2020, representing 44 cases (13.8%), with similar proportions at Hospital A (11.9%) and Hospital B (15.6%). The relatively modest proportion of COVID-19 cases reflects our inclusion criteria, which required antibiotic prescription for respiratory infection; many COVID-19 patients were managed without antibiotics, consistent with guidance discouraging empirical antibiotic use in viral infection.

The study population had substantial comorbidity burden. Hypertension was the most prevalent comorbidity (291 patients, 45.5%), followed by diabetes mellitus (119 patients, 18.6%), atrial fibrillation (125 patients, 19.5%), and chronic kidney disease (121 patients, 18.9%). Notable changes during the pandemic period included increased prevalence of heart failure (32 [10.0%] in 2019 vs 63 [19.7%] in 2020, *p* < 0.001) and decreased chronic kidney disease (75 [23.4%] vs 46 [14.4%], *p* = 0.003).

### Antibiotic classification by WHO AWaRe categories

**Figure 2.**
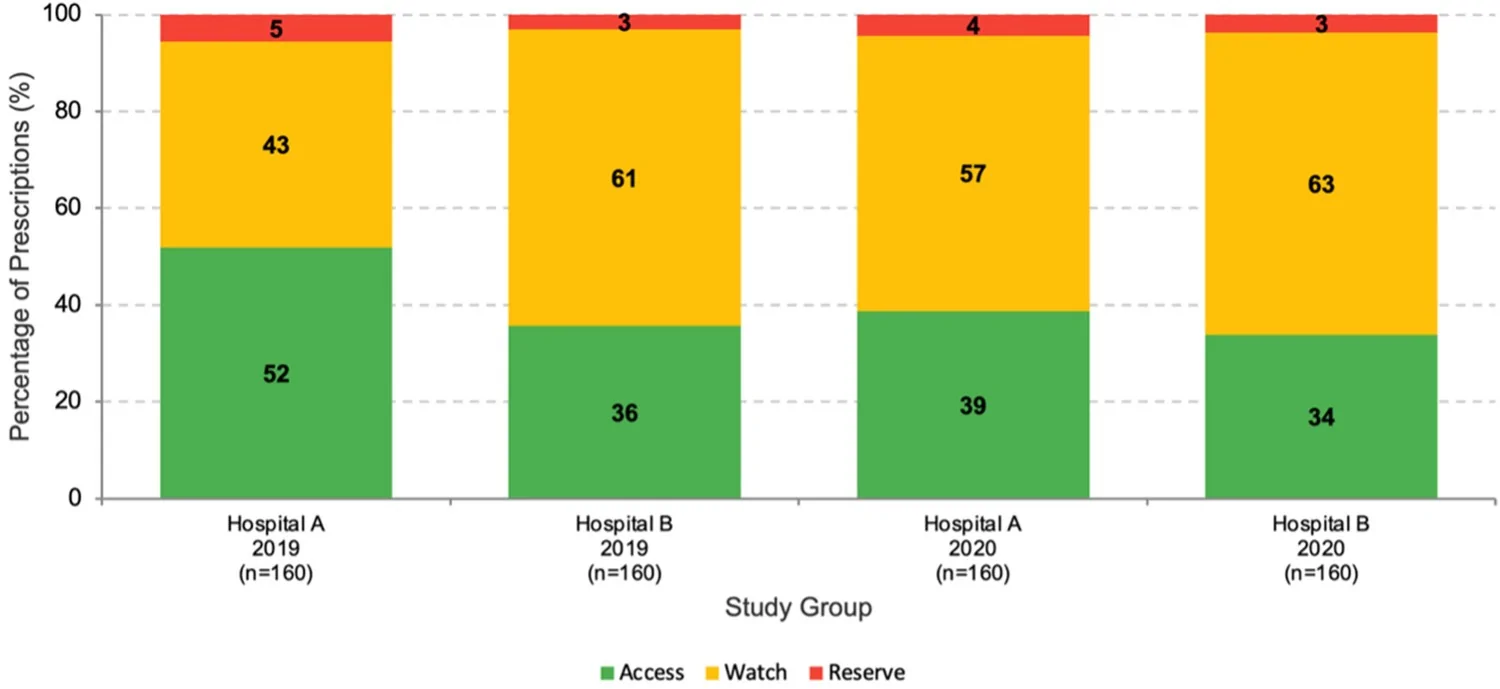
WHO AWaRe antibiotic classification by hospital and study period. Note: Stacked bar chart illustrating the distribution of empirical antibiotic prescriptions according to the World Health Organisation AWaRe (Access, Watch, Reserve) classification across two hospitals during pre-pandemic (2019) and pandemic (2020) periods. Sample size: *n* = 160 patients per group (N = 640 total). Access antibiotics (green) represent first- and second-choice agents for common infections with lower resistance potential; Watch antibiotics (amber) represent agents with higher resistance potential, recommended only for specific, limited indications; Reserve antibiotics (red) represent last-resort agents reserved for treatment of confirmed or suspected multidrug-resistant infections. Percentages are rounded to whole numbers and sum to 100% for each study group. A shift toward increased Watch antibiotic use was observed across all groups during 2020, with corresponding reductions in Access antibiotic prescribing. *P* < 0.001 (chi-square test comparing all four groups). Abbreviations: AWaRe, Access, Watch, Reserve; WHO, World Health Organisation.

Figure 2 presents antibiotic prescribing patterns according to the WHO AWaRe classification. Watch category antibiotics were the most frequently prescribed overall, with utilisation increasing from 52% (166/320) in 2019–60% (191/320) in 2020 (*p* < 0.001). A notable shift occurred at Hospital A, where prescribing changed from predominantly Access antibiotics (52%) in 2019 to Watch antibiotics (57%) in 2020, representing a 14 percentage-point increase in Watch antibiotic use. Hospital B consistently demonstrated higher Watch antibiotic utilisation than Hospital A during both periods (61% vs 43% in 2019; 63% vs 57% in 2020). Access antibiotic prescribing declined across both hospitals, from 52% to 39% at Hospital A and from 36% to 34% at Hospital B. Reserve antibiotics remained appropriately restricted across all groups (3–5%), with 14 prescriptions (4.4%) in 2019 and 13 prescriptions (4.1%) in 2020, indicating sustained adherence to stewardship principles for last-resort agents despite pandemic pressures. The most commonly prescribed antibiotics by AWaRe category are listed in Supplemental Table S3.

### Antibiotic review timing

**Figure 3.**
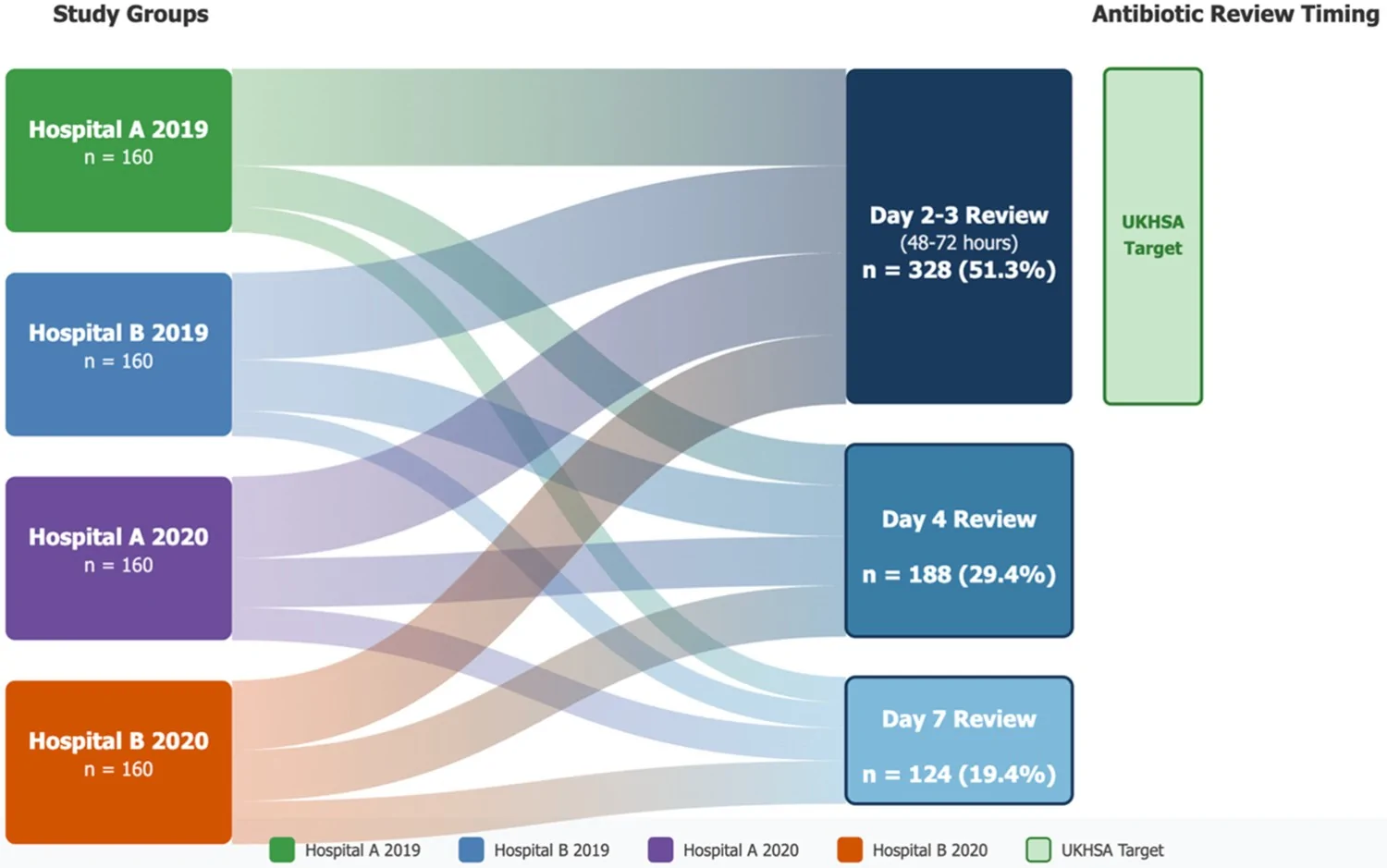
Antibiotic review timing by hospital and study period (Sankey diagram, N = 640). Note: Sankey diagram illustrating the flow of antibiotic prescriptions from four study groups to documented review timing categories. Left nodes represent study groups: Hospital A 2019 (green), Hospital B 2019 (blue), Hospital A 2020 (purple), and Hospital B 2020 (orange), each comprising 160 patients. Right nodes indicate timing of first documented antibiotic review: Day 2–3 Review (48–72 h), *n* = 328 (51.3%); Day 4 Review, *n* = 188 (29.4%); Day 7 Review, *n* = 124 (19.4%). Flow width is proportional to the number of patients from each study group reaching each review timing category. The UKHSA Target indicator denotes the recommended standard for antibiotic review within 48–72 h of initiation. Note: Review timing was determined from the first documented clinical review that included explicit assessment of antibiotic appropriateness, recorded in medical notes or electronic prescribing systems. Abbreviations: UKHSA, UK Health Security Agency.

Figure 3 illustrates the timing of antibiotic reviews across hospitals and study periods. Early review (Day 2–3) was the predominant practice, occurring in 328 patients (51.3%) overall. Day 4 reviews accounted for 188 patients (29.4%), while Day 7 reviews occurred in 124 patients (19.4%).

Early review rates were similar between study periods: 165 patients (51.6%) in 2019 versus 163 patients (50.9%) in 2020 (*p* = 0.87). However, hospital-level patterns showed temporal variation. Hospital B demonstrated a shift from predominantly early reviews in 2019 (54.4%) toward more Day 4 reviews in 2020 (34.4% vs 26.9% in 2019, *p* = 0.14), while Hospital A showed the opposite pattern, with increased early review rates during the pandemic (53.8% vs 48.8%, *p* = 0.36).

Combined early and intermediate reviews (Day 2–3 and Day 4) accounted for 80.6% of all antibiotic reviews, indicating effective implementation of guideline-recommended antibiotic review practices at both hospitals despite pandemic-related operational challenges in both 2019 and 2020.

### Antimicrobial stewardship interventions

**Figure 4.**
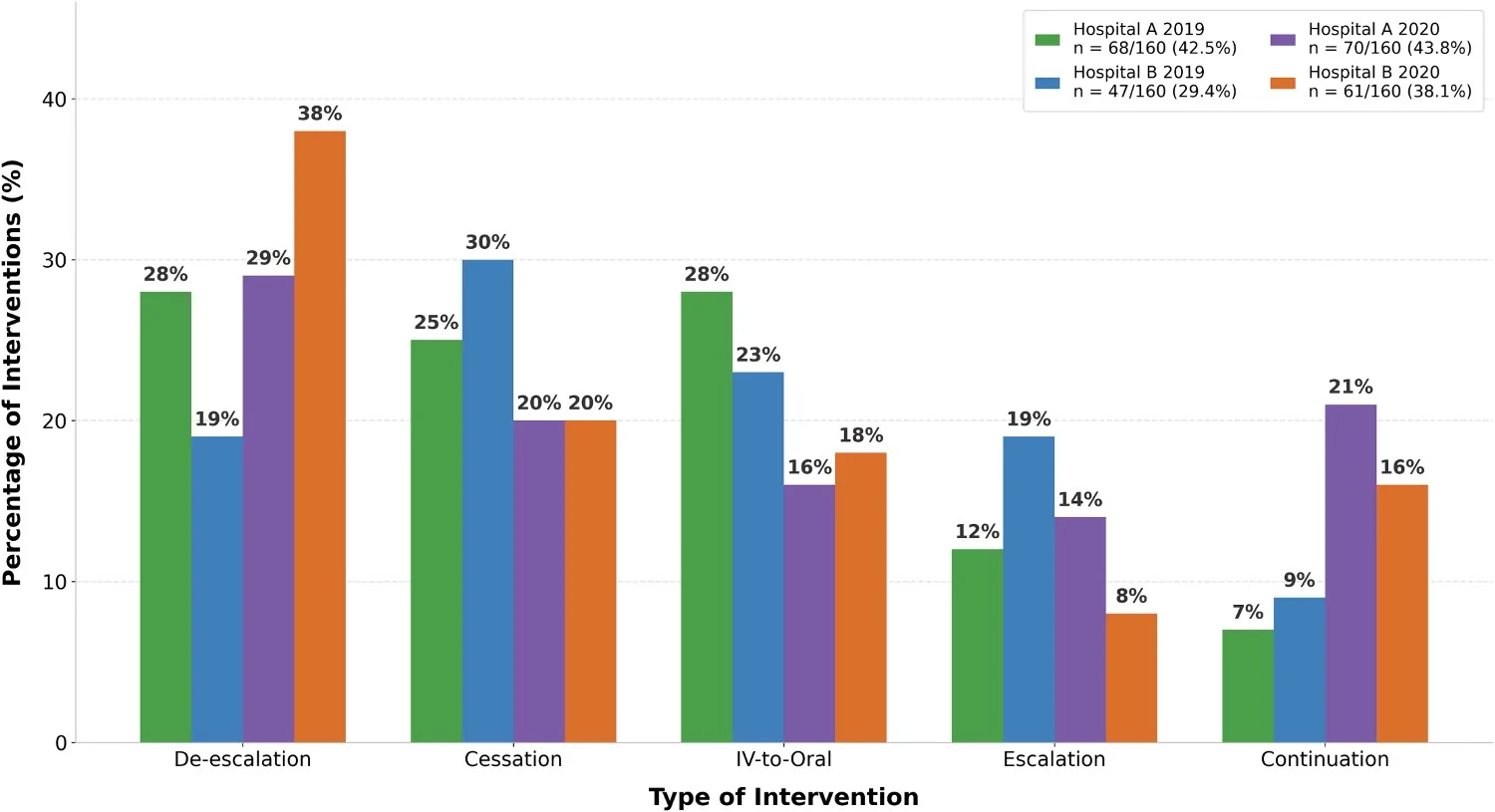
Distribution of antimicrobial stewardship interventions among guideline-concordant prescriptions by hospital and study period. Note: Bar chart illustrating the percentage of antimicrobial stewardship interventions implemented following guideline-concordant initial antibiotic prescriptions across two hospitals during pre-pandemic (2019) and pandemic (2020) periods. Sample sizes: Hospital A 2019, *n* = 68/160 (42.5%); Hospital B 2019, *n* = 47/160 (29.4%); Hospital A 2020, *n* = 70/160 (43.8%); Hospital B 2020, *n* = 61/160 (38.1%). Note: Denominators (n/160) represent patients with guideline-concordant initial antibiotic prescribing, defined as empirical antibiotic selection aligned with Trust antimicrobial guidelines based on clinical indication, infection severity, and patient-specific factors. De-escalation, switch to narrower-spectrum antibiotic; Cessation, antibiotic discontinued; IV-to-Oral, intravenous to oral route conversion; Escalation, switch to broader-spectrum antibiotic; Continuation, no change with documented review.

Figure 4 presents antimicrobial stewardship interventions among patients whose initial antibiotic prescription was concordant with local guidelines (*N* = 246). Among the remaining guideline-discordant prescriptions, stewardship actions documented included prescriber feedback, dose optimisation, and in some cases cessation or de-escalation following pharmacist or microbiologist review; however, the small numbers precluded formal subgroup analysis and these are acknowledged as a limitation. De-escalation to narrower-spectrum agents was a frequent intervention, with Hospital B 2020 demonstrating the highest rate (38%), followed by Hospital A 2020 (29%), Hospital A 2019 (28%), and Hospital B 2019 (19%). Antibiotic cessation was most frequent at Hospital B in 2019 (30%) and Hospital A in 2019 (25%), declining to 20% at both hospitals in 2020. Intravenous-to-oral switch rates were notably higher in 2019 (Hospital A: 28%; Hospital B: 23%) compared to 2020 (Hospital A: 16%; Hospital B: 18%), potentially reflecting reduced opportunities for early discharge planning during the pandemic. Escalation to broader-spectrum therapy remained relatively uncommon across all groups (8–19%). Notably, continuation of antibiotic therapy without documented change increased substantially during the pandemic period, rising from 7% to 21% at Hospital A and from 9% to 16% at Hospital B between 2019 and 2020. This pattern suggests more conservative prescribing behaviour during COVID-19, with clinicians potentially opting to maintain existing antibiotic regimens rather than implement de-escalation or early cessation strategies amid clinical uncertainty.

## Discussion

### Principal findings

This retrospective cohort study of 640 patients across two UK hospitals demonstrates that antimicrobial stewardship practices, particularly early antibiotic review, remained resilient during the COVID-19 pandemic despite significant shifts in prescribing patterns. Early reviews (Day 2–3) were maintained at approximately 50% of assessments during both study periods, enabling continued stewardship interventions, including de-escalation and antibiotic cessation. However, the pandemic was associated with a marked shift toward Watch category antibiotics, with prescribing increasing from 55.0% to 63.1%. This is notable given the WHO global target recommending that at least 60% of antibiotic consumption should be from the Access category, a threshold not met in either study period at either hospital (WHO, 2023).

### Antibiotic review timing in context

The predominance of early antibiotic reviews (48.1–54.4% at Day 2–3) aligns with international stewardship guidelines recommending reassessment within 48–72 h when diagnostic results become available. This timing aligns with the UK Health Security Agency's ‘Start Smart – Then Focus’ framework, which mandates active review of all patients on antibiotics 48 h after initiation (UKHSA, 2024). Our findings are consistent with the ARK-Hospital stepped-wedge cluster-randomised trial, which demonstrated that systematic antibiotic review interventions achieved sustained reductions in antibiotic consumption (−4.8% annually) without compromising patient safety (Llewelyn et al., 2022). Notably, the Antibiotic Review Kit (ARK) trial in hospitals identified similar patterns of review timing and intervention types to those observed in our study, validating the generalisability of structured review approaches across UK hospital settings (ARK, 2024).

The maintenance of early review rates during the pandemic period is particularly significant given documented disruptions to stewardship activities elsewhere. A qualitative study of Scottish acute care hospitals reported substantial workforce redeployment and reduced multidisciplinary engagement during COVID-19, suggesting our findings may reflect institutional resilience or established stewardship infrastructure (Matuluko et al., 2024). The English Surveillance Programme for Antimicrobial Utilisation and Resistance (ESPAUR) report in 2024 highlighted ongoing challenges in maintaining stewardship activities during the pandemic recovery period, emphasising the importance of embedded review processes that can withstand operational pressures (ESPAUR, 2024).

### AWaRe classification and prescribing patterns

The observed shift toward Watch category antibiotics during the pandemic reflects a pattern documented globally (Nandi et al., 2023). Our finding that Watch antibiotic use increased from 55.0% to 63.1% is consistent with systematic reviews demonstrating increased broad-spectrum prescribing during COVID-19, driven by diagnostic uncertainty and concerns about bacterial co-infection (Abdelsalam Elshenawy et al., 2023). However, evidence suggests that true bacterial co-infection rates in COVID-19 patients were substantially lower than empirical antibiotic prescribing rates would suggest, approximately 8% compared with antibiotic prescription rates exceeding 70% (Alshaikh et al., 2022; Rothe et al., 2021).

The institutional variation observed between hospitals, with Hospital B consistently demonstrating higher Watch antibiotic utilisation (63.8–65.0% vs 46.3–61.2%), warrants attention. Such variation may reflect differences in local resistance patterns, patient case-mix, or prescribing culture. The 2024 ESPAUR report documented similar regional and institutional variation in antibiotic consumption across England, highlighting the importance of local stewardship benchmarking (ESPAUR, 2024). Encouragingly, Reserve antibiotic use remained appropriately restricted (<2%) across all groups, indicating that stewardship policies for last-resort agents were maintained throughout the pandemic.

### Stewardship interventions and optimisation opportunities

De-escalation emerged as the predominant stewardship intervention (19.1–37.7%), consistent with the CARES framework emphasising therapy optimisation following clinical review (UKHSA, 2024). The increased de-escalation activity during the pandemic period, particularly at Hospital B (19.1% to 37.7%), may reflect heightened awareness of antimicrobial resistance risks or improved diagnostic certainty following initial empirical therapy. This pattern aligns with evidence demonstrating that systematic approaches to therapy review can identify substantial opportunities for antibiotic optimisation even when initial prescribing adheres to guidelines (Llor et al., 2024; Wilkinson et al., 2018).

Antibiotic cessation rates (19.7–29.8%) support the ‘cease’ component of the CARES framework, indicating that clinicians actively discontinued therapy when infection was not confirmed. The British Society for Antimicrobial Chemotherapy (BSAC) advocates for such ‘back-end’ stewardship strategies, prospective audit and feedback following prescription, as more clinically acceptable and effective than restrictive front-end approaches (BSAC, 2015). The observed decline in cessation rates during the pandemic (from 25–30% in 2019–20% in 2020) is consistent with findings from comparable hospital studies reporting reduced antibiotic discontinuation during COVID-19 owing to diagnostic uncertainty and heightened caution regarding bacterial co-infection (Pierce & Stevens, 2021; Rothe et al., 2021). Our findings support this evidence-based model.

### Pharmacist contribution to stewardship resilience

The maintenance of stewardship activities during the pandemic period reflects the critical contribution of the pharmacy workforce. A concurrent survey of pharmacists at the same NHS Foundation Trust revealed that 79.2% valued enhanced communication with microbiologists and AMS teams during COVID-19, and 64% adhered to stewardship principles by discontinuing antibiotics following negative cultures (Abdelsalam Elshenawy et al., 2025). Furthermore, 86.4% of pharmacists agreed that antimicrobial misuse during the pandemic could impact resistance, demonstrating strong awareness of AMR implications. These findings suggest that pharmacist engagement was associated with sustained early review practices and the implementation of de-escalation interventions despite operational pressures. The adoption of technology-enabled stewardship, supported by 63.2% of pharmacists through virtual multidisciplinary meetings, facilitated continued stewardship co-ordination and maintained team communication across clinical settings during the pandemic. These survey findings are associative and should be interpreted cautiously; direct causal attribution of stewardship outcomes to pharmacist activities was not possible within the current study design.

### Clinical implications and recommendations

The findings from this study have important implications for clinical practice and policy across multiple domains. For clinical teams, early antibiotic review within 48–72 h should be embedded as a mandatory step in clinical pathways for all patients receiving empirical antibiotic therapy. Our data demonstrate that such reviews enable meaningful stewardship interventions, including de-escalation in approximately one-quarter of appropriately prescribed cases, without compromising patient outcomes. Clinical teams should document review decisions using structured frameworks such as CARES to facilitate audit and feedback, ensuring that stewardship actions are visible, measurable, and sustainable over time.

For stewardship programmes, AWaRe classification provides actionable metrics for monitoring prescribing patterns and benchmarking institutional performance. The observed variation between hospitals suggests opportunities for peer comparison and shared learning within NHS Trusts. Stewardship teams should monitor Watch antibiotic consumption as a key performance indicator, with targets aligned to WHO recommendations. Pharmacists occupy a pivotal position in antimicrobial stewardship delivery, bridging prescribing decisions with therapeutic outcomes through prospective audit, direct prescriber engagement, and patient counselling. Evidence from pharmacist surveys at the study sites demonstrated that 80% reported COVID-19 patient conditions influenced antibiotic prescribing decisions, highlighting the need for pharmacist involvement in complex clinical decision-making. Building programme resilience requires diversifying stewardship delivery models to include pharmacist-led ward rounds, remote prescription review, and automated decision support, ensuring continuity when individual components are disrupted. The expansion of pharmacist prescribing rights and integration of clinical pharmacists into ward-based teams presents opportunities for sustainable, embedded stewardship. As the Royal Pharmaceutical Society emphasises, pharmacist leadership within multidisciplinary teams, combined with structured AMS training, is essential for empowering pharmacists to co-lead stewardship initiatives and address AMR challenges effectively.

For pandemic preparedness, our findings demonstrate that core stewardship activities can be maintained during health emergencies when review processes are embedded in routine clinical workflows. Future pandemic planning should explicitly protect stewardship infrastructure, including dedicated pharmacist and microbiologist time, electronic prescribing prompts, and audit capacity. The shift toward broader-spectrum prescribing during COVID-19 highlights the need for rapid diagnostic development to reduce empirical antibiotic use during future outbreaks.

For sustainability and resilience, healthcare systems must recognise antimicrobial stewardship as essential infrastructure rather than an optional enhancement. Sustainable stewardship requires dedicated, protected funding for specialist personnel; integration of stewardship metrics into organisational quality frameworks; and succession planning to maintain institutional expertise. Resilient programmes are characterised by distributed leadership, cross-training among team members, and flexible delivery models that can adapt to workforce constraints. The pandemic demonstrated that programmes relying solely on face-to-face interventions were vulnerable to disruption, whereas those with embedded electronic systems and multidisciplinary ownership maintained effectiveness. Investment in digital infrastructure, including electronic prescribing with integrated decision support, automated surveillance dashboards, and remote review capabilities, provides both sustainability through reduced reliance on individual practitioners and resilience through continued functionality during crises.

For policy, integration of electronic antibiotic review prompts within prescribing systems could sustain stewardship during periods of workforce pressure. National surveillance programmes should continue monitoring AWaRe consumption patterns to track recovery from pandemic-related prescribing shifts and progress toward WHO targets. Policymakers should consider mandating antimicrobial stewardship programme accreditation standards that include resilience assessments, ensuring healthcare organisations can maintain effective stewardship across varying operational conditions.

### Strengths and limitations

This study has several strengths. The sample of 640 patients across two hospitals and two time periods enabled comparison of stewardship practices before and during the pandemic. Application of established frameworks (UKHSA Start Smart – Then Focus, CARES, and WHO AWaRe) facilitates benchmarking against national and international standards. The detailed assessment of antibiotic review timing and intervention types provides practical insights for stewardship implementation. Rigorous data quality measures, including 5% independent double extraction with substantial inter-rater agreement (κ = 0.84–0.91), enhance reliability. Adherence to STROBE guidelines supports transparent reporting.

Several limitations should be acknowledged. The retrospective design introduces potential documentation bias; antibiotic reviews may have occurred but not been recorded. Both hospitals were within a single NHS Foundation Trust, potentially limiting generalisability to other healthcare settings. The stratified sampling approach, while ensuring temporal representation, means that findings reflect proportions within our sample rather than the true population prevalence. The absence of microbiological culture data limits the assessment of prescribing appropriateness beyond guideline concordance. Low chest radiography rates (29.1% overall) may reflect institutional practice variation that influenced empirical prescribing decisions. The purely descriptive analysis with post-hoc statistical comparisons limits causal inference; observed differences between periods and hospitals should be interpreted as associations requiring further investigation. Additionally, the study did not adjust for potential confounders such as illness severity, COVID-19 diagnosis, or patient comorbidity burden, which may have influenced prescribing patterns independently of stewardship activities. Finally, review timing classification may be subject to misclassification bias, as documentation practices varied across clinical teams and some antibiotic reviews may have occurred without formal recording in the electronic health record.

## Conclusion

This study demonstrates that early antibiotic review practices can be maintained during healthcare crises, enabling continued stewardship interventions despite operational pressures. Day 2–3 reviews facilitated de-escalation and therapy cessation consistent with the UKHSA Start Smart – Then Focus framework. However, the shift toward Watch category antibiotics during the pandemic highlights the need for enhanced diagnostic stewardship to reduce empirical broad-spectrum prescribing. Pharmacists play a pivotal role in driving sustainable antimicrobial stewardship, serving as the bridge between evidence-based guidelines and bedside implementation. Concurrent workforce surveys demonstrated exemplary pharmacist knowledge of AMR and strong support for stewardship activities, indicating capacity to co-lead AMS initiatives during health emergencies. Enhanced training and workforce development are essential to empower pharmacists in confronting AMR and safeguarding patient lives. Future antimicrobial stewardship programmes should prioritise embedded review processes, institutional benchmarking using AWaRe metrics, and pandemic-resilient infrastructure to sustain effective antibiotic optimisation during health system disruptions.

## Supplementary Material

Graphic Abstract jpeg.png

Supplementary Table S2.docx

Supplementary Table S1.docx

Supplementary Table S3.docx

STROBE Checklist.docx

Copyright Permissions.pdf

## Data Availability

The data sets generated during the current study are available from the corresponding author upon reasonable request.
