# Supplementary material for: Early antibiotic review and AWaRe prescribing in respiratory infections before vs during COVID-19: a two-hospital retrospective cohort study
Source: J Pharm Policy Pract. 2026 Sep 14;19(1):2719528. doi: 10.1080/20523211.2026.2719528 (PMC13577274; doi:10.1080/20523211.2026.2719528)
Supplement: Supplementary file 1 [file JPPP_A_2719528_SM7213.pdf]

Permissions and Third-Party Material Declaration

Manuscript title:
Sustaining Antimicrobial Stewardship in Hospitalised Patients: A Comparative Analysis of Early Antibiotic Review and Prescribing Trends Over Two Years

Declaration:
The authors confirm that no third-party copyrighted material has been used in this manuscript. All figures, tables, diagrams, and supplementary materials were created by the authors specifically for this study. No previously published figures or tables have been reproduced. No copyrighted tools, scales, or instruments beyond fair use have been included.

Accordingly, no permissions or licences were required for this submission.

Signed on behalf of all authors,

Corresponding Author

Dr Rasha Abdelsalam Elshenawy

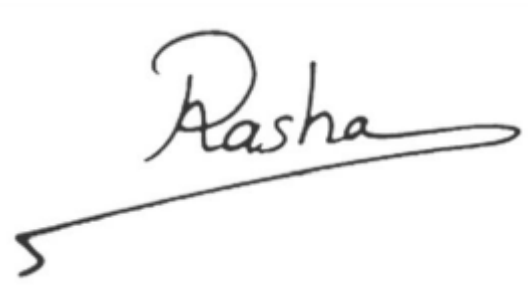

School of Health, Medicine and Life Sciences
University of Hertfordshire
Hatfield, United Kingdom
Email: r.elshenawy@herts.ac.uk

29 December 2025
