# Supplementary material for: Early antibiotic review and AWaRe prescribing in respiratory infections before vs during COVID-19: a two-hospital retrospective cohort study
Source: J Pharm Policy Pract. 2026 Sep 14;19(1):2719528. doi: 10.1080/20523211.2026.2719528 (PMC13577274; doi:10.1080/20523211.2026.2719528)
Supplement: Supplementary file 2 [file JPPP_A_2719528_SM7214.docx]

**STROBE Checklist (Retrospective cohort study)**

*Sustaining Antimicrobial Stewardship in Hospitalised Patients: A Comparative Analysis of Early Antibiotic Review and Prescribing Trends Over Two Years*

**How to use:** Provide the section heading (and subheading) where each STROBE item is addressed. If your submission portal requests page numbers, replace the section references below with page numbers after final formatting.

| Item | STROBE recommendation (combined checklist) | Where addressed in this manuscript |
| --- | --- | --- |
| 1a | Indicate the study’s design with a commonly used term in the title or the abstract. | Title; Abstract (Methods). |
| 1b | Provide in the abstract an informative and balanced summary of what was done and what was found. | Abstract (Background/Methods/Results/Conclusions). |
| 2 | Explain the scientific background and rationale for the investigation being reported. | Introduction (AMR burden; AMS and antibiotic review rationale). |
| 3 | State specific objectives, including any prespecified hypotheses. | Introduction (final paragraph: study aims/objectives). |
| 4 | Present key elements of study design early in the paper. | Methods – Study Design and Setting; Sampling Strategy. |
| 5 | Describe the setting, locations, and relevant dates, including periods of recruitment, exposure, follow-up, and data collection. | Methods – Study Design and Setting; Study periods (2019 and 2020) and time-points (March/June/Sept/Dec). |
| 6a | Give the eligibility criteria, and the sources and methods of selection of participants. | Methods – Study Population and Eligibility Criteria; Sampling Strategy; ICD-10 identification. |
| 6b | For matched studies, give matching criteria and number of exposed and unexposed (if applicable). | Not applicable (no matching). |
| 7 | Clearly define all outcomes, exposures, predictors, potential confounders, and effect modifiers. Give diagnostic criteria, if applicable. | Methods – Data Sources and Extraction (variables; outcomes; appropriateness definition; review timing categories). |
| 8 | For each variable of interest, give sources of data and details of methods of assessment (measurement). Describe comparability of assessment methods if more than one group. | Methods – Data Sources and Extraction; Data Quality and Validation (double extraction; kappa). |
| 9 | Describe any efforts to address potential sources of bias. | Methods – Sampling Strategy (systematic random sampling); Data Quality and Validation; Limitations (documentation bias). |
| 10 | Explain how the study size was arrived at. | Methods – Sample Size Calculation. |
| 11 | Explain how quantitative variables were handled in the analyses. If applicable, describe which groupings were chosen and why. | Methods – Review timing categories (Day 2–3/Day 4/Day 7); Statistical Analysis. |
| 12a | Describe all statistical methods, including those used to control for confounding. | Methods – Statistical Analysis (chi-square/Fisher’s exact; Mann–Whitney U; p<0.05). |
| 12b | Describe any methods used to examine subgroups and interactions. | Results – Stratified comparisons by hospital and period (Figures 2–4; text). |
| 12c | Explain how missing data were addressed. | Not stated explicitly (add a sentence in Methods if missing data occurred; otherwise state ‘no missing data for key variables’). |
| 12d | If applicable, explain how loss to follow-up was addressed. | Not applicable (retrospective inpatient cohort; outcomes captured during admission). |
| 12e | Describe any sensitivity analyses. | Not reported (optional—add if performed). |
| 13a | Report numbers of individuals at each stage of study. | Results – Study Population; Figure 1 (Patient flow diagram). |
| 13b | Give reasons for non-participation at each stage. | Methods – Exclusion criteria; Results/Figure 1 (where reported). |
| 13c | Consider use of a flow diagram. | Figure 1 (Patient Selection Flow Diagram). |
| 14a | Give characteristics of study participants and information on exposures and potential confounders. | Results – Table 1 (demographics, indications, comorbidities, outcomes). |
| 14b | Indicate number of participants with missing data for each variable of interest. | Not stated explicitly (add to Results if applicable). |
| 15 | Report numbers of outcome events or summary measures over time. | Results – Mortality/discharge; prescribing patterns and review timing (Table 1; Figures 2–4). |
| 16a | Give unadjusted estimates and, if applicable, confounder-adjusted estimates and their precision (e.g., 95% CI). | Results – Group comparisons with p-values; no adjusted models reported. |
| 16b | Report category boundaries when continuous variables were categorized. | Methods – Review timing categories (Day 2–3; Day 4; Day 7). |
| 16c | If relevant, consider translating relative risk into absolute risk for a meaningful time period. | Not applicable / not reported (optional). |
| 17 | Report other analyses done—e.g., analyses of subgroups and interactions, and sensitivity analyses. | Results – Hospital-level analyses; guideline-concordant subgroup (Figure 4). |
| 18 | Summarise key results with reference to study objectives. | Discussion – Principal Findings; Review timing; AWaRe patterns. |
| 19 | Discuss limitations of the study, taking into account sources of potential bias or imprecision. | Discussion – Strengths and Limitations. |
| 20 | Give a cautious overall interpretation considering objectives, limitations, and other evidence. | Discussion – Interpretation with literature comparison; implications. |
| 21 | Discuss the generalisability (external validity) of the study results. | Discussion – Strengths and Limitations (single Trust; generalisability). |
| 22 | Give the source of funding and the role of the funders. | End matter – Funding; Competing interests. |

**Reference:** STROBE Statement checklist (combined cohort/case-control/cross-sectional) – STROBE initiative / EQUATOR Network.
