# Supplementary material for: Early antibiotic review and AWaRe prescribing in respiratory infections before vs during COVID-19: a two-hospital retrospective cohort study
Source: J Pharm Policy Pract. 2026 Sep 14;19(1):2719528. doi: 10.1080/20523211.2026.2719528 (PMC13577274; doi:10.1080/20523211.2026.2719528)
Supplement: Supplementary file 3 [file JPPP_A_2719528_SM7215.docx]

**Supplementary Table S3:** Antibiotics prescribed by WHO AWaRe classification category

| **AWaRe Category** | **Antibiotics Prescribed** | **Description** |
| --- | --- | --- |
| **ACCESS** | Amoxicillin  Ampicillin  Benzylpenicillin  Doxycycline  Metronidazole  Trimethoprim  Nitrofurantoin | First-line antibiotics for common infections. These antibiotics have a relatively narrow spectrum of activity and lower potential for resistance development. Should be widely available, affordable, and quality-assured. |
| **WATCH** | Co-amoxiclav (amoxicillin-clavulanate)  Piperacillin-tazobactam  Clarithromycin  Azithromycin  Ciprofloxacin  Levofloxacin  Moxifloxacin  Ceftriaxone  Cefuroxime  Ceftazidime  Meropenem  Ertapenem  Gentamicin  Vancomycin | Antibiotics with higher resistance potential. These should be prioritised as key targets for stewardship programmes and monitoring. Recommended only for specific, limited indications. Includes most critically important antimicrobials such as fluoroquinolones, third-generation cephalosporins, macrolides, and carbapenems. |
| **RESERVE** | Linezolid  Colistin  Daptomycin  Tigecycline | Last-resort antibiotics reserved for treatment of confirmed or suspected infections due to multidrug-resistant organisms. Use should be restricted to highly specific patients and settings when all other alternatives have failed or are unsuitable. Subject to strict stewardship oversight. |

**Study Prescribing Patterns:**

| **AWaRe Category** | **Hospital A 2019** | **Hospital B 2019** | **Hospital A 2020** | **Hospital B 2020** |
| --- | --- | --- | --- | --- |
| **Access** | 83 (51.9%) | 57 (35.6%) | 62 (38.8%) | 54 (33.8%) |
| **Watch** | 74 (46.3%) | 102 (63.8%) | 98 (61.2%) | 104 (65.0%) |
| **Reserve** | 3 (1.9%) | 1 (0.6%) | 0 (0.0%) | 2 (1.3%) |

*Source: WHO AWaRe Classification of Antibiotics for Evaluation and Monitoring of Use, 2023. Geneva: World Health Organisation; 2023 (WHO/MHP/HPS/EML/2023.04).*

**Note:** The WHO global target recommends that at least 60% of total antibiotic consumption should be from the Access category. In this study, only Hospital A in 2019 approached this target (51.9%).
