# Supplementary material for: Early antibiotic review and AWaRe prescribing in respiratory infections before vs during COVID-19: a two-hospital retrospective cohort study
Source: J Pharm Policy Pract. 2026 Sep 14;19(1):2719528. doi: 10.1080/20523211.2026.2719528 (PMC13577274; doi:10.1080/20523211.2026.2719528)
Supplement: Supplementary file 4 [file JPPP_A_2719528_SM7216.docx]

**Supplementary Table S1:** ICD-10 codes used for identification of patients with respiratory tract infections

| **Diagnosis** | **ICD-10 Code(s)** |
| --- | --- |
| Community-acquired pneumonia (CAP) | J13, J14, J15, J16, J18 |
| Hospital-acquired pneumonia (HAP) | J18.9 (with hospital admission >48 hours prior to diagnosis) |
| Ventilator-associated pneumonia (VAP) | J95.851 |
| COPD with acute exacerbation | J44.0, J44.1 |
| Lower respiratory tract infection (LRTI) | J20, J21, J22 |
| Upper respiratory tract infection (URTI) | J00, J01, J02, J03, J04, J05, J06 |
| Unspecified pneumonia | J18.9 |
| COVID-19 pneumonia | U07.1, U07.2 |

**ICD-10 Code Descriptions:**

*J13 – Pneumonia due to Streptococcus pneumoniae*

*J14 – Pneumonia due to Haemophilus influenzae*

*J15 – Bacterial pneumonia, not elsewhere classified*

*J16 – Pneumonia due to other infectious organisms, not elsewhere classified*

*J18 – Pneumonia, unspecified organism*

*J20 – Acute bronchitis*

*J21 – Acute bronchiolitis*

*J22 – Unspecified acute lower respiratory infection*

*J00–J06 – Acute upper respiratory infections (includes acute nasopharyngitis, sinusitis, pharyngitis, tonsillitis, laryngitis)*

*J44.0 – COPD with acute lower respiratory infection*

*J44.1 – COPD with acute exacerbation*

*J95.851 – Ventilator-associated pneumonia*

*U07.1 – COVID-19, virus identified*

*U07.2 – COVID-19, virus not identified (clinically-epidemiologically diagnosed)*

*Source: International Classification of Diseases, Tenth Revision (ICD-10), World Health Organization, 2019 version.*
