# Supplementary material for: Early antibiotic review and AWaRe prescribing in respiratory infections before vs during COVID-19: a two-hospital retrospective cohort study
Source: J Pharm Policy Pract. 2026 Sep 14;19(1):2719528. doi: 10.1080/20523211.2026.2719528 (PMC13577274; doi:10.1080/20523211.2026.2719528)
Supplement: Supplementary file 5 [file JPPP_A_2719528_SM7217.docx]

**Supplementary Table S2.** Data Sources and Variables Extracted

| **Category** | **Variables / Definitions** |
| --- | --- |
| **Demographics** | Age, Sex |
| **Clinical Characteristics** | Primary diagnosis, Comorbidities*, Documented drug allergies |
| **Prescribing Data** | Initial empirical antibiotic, Antibiotic class (WHO AWaRe classification: Access, Watch, Reserve), Route of administration |
| **Diagnostic Findings** | Chest radiograph results: Pneumonia confirmed, No pneumonia, Not performed |
| **Antibiotic Review Timing** | Day 2-3 (Early), Day 4 (Intermediate), Day 7 (Late)  *Aligned with UKHSA Start Smart – Then Focus framework* |
| **Stewardship Interventions** | Continuation, De-escalation, Escalation, IV-to-oral switch, Cessation  *Categorised per UKHSA AMS Toolkit / CARES outcomes* |
| **Appropriate Prescribing** | Concordance with Trust's local antimicrobial guidelines for empirical therapy, based on:  Clinical indication, Infection severity, Patient factors (allergies, renal function), Documented rationale  *Classification: Appropriate or Inappropriate* |
| **Outcomes** | Discharge, In-hospital mortality |

***Comorbidities defined as:**

Hypertension (documented diagnosis or antihypertensive therapy); Diabetes mellitus (Type 1 or 2); Chronic kidney disease (Stage 3-5 or AKI); Chronic liver disease (cirrhosis, hepatitis, or fatty liver disease); Heart failure (with or without reduced ejection fraction); Malignancy (active cancer); Chronic obstructive pulmonary disease (COPD)

**Abbreviations:** *AWaRe = Access, Watch, Reserve; UKHSA = UK Health Security Agency; AMS = Antimicrobial Stewardship; CARES = Cease, Amend, Review, Escalate, Switch; AKI = Acute Kidney Injury*
